# Effects of Pasture Grass, Silage, and Hay Diet on Equine Fecal Microbiota

**DOI:** 10.3390/ani11051330

**Published:** 2021-05-07

**Authors:** Yiping Zhu, Xuefan Wang, Liang Deng, Shulei Chen, Chunyan Zhu, Jing Li

**Affiliations:** 1 Equine Clinical Diagnostic Center, College of Veterinary Medicine, China Agricultural University, Beijing 100193, China; yipingz@cau.edu.cn (Y.Z.); xuefan.wang@foxmail.com (X.W.); shuleichen20@163.com (S.C.); 2 College of Animal Husbandry and Veterinary Medicine, Shenyang Agricultural University, Shenyang 110866, China; ldeng@syau.edu.cn; 3 Shanghai Center of Agri-Products Quality and Safety, Shanghai 201708, China; suosuo152@hotmail.com

**Keywords:** fecal microbiota, hay, horses, pasture, silage, 16S rRNA

## Abstract

**Simple Summary:**

The intestinal microbial community in horses is very complex and interacts closely with diets. Apart from traditional forage diets, such as hay and pasture grass, silage is used to feed horses in China and other areas of the world for economic reasons or convenience of storage. Additionally, silage is also used for its convenience of harvesting and its nutrient components, including lactic acid and volatile fatty acids. In this study, we detected the characteristic composition of a fecal microbial community in horses that were fed silage with the use of a relatively new sequencing technique; we compared this result with that from horses that were fed hay and pasture grass. This study revealed some characteristic findings on the fecal microbial composition in horses that were given each of type of diet and showed significant differences between the groups. Our results provided novel data about the fecal microbial composition in horses on the silage diet. We hope that these could help balance the intestinal microbiota in horses that are mainly fed silage in combination with other types of forages in order to maintain intestinal health.

**Abstract:**

Diet is an important factor affecting intestinal microbiota in horses. Fecal microbiota is commonly used as a substitute for studying hindgut microbiota when investigating the relationship between intestinal microbial changes and host health. So far, no study has compared the difference between the fecal microbiota found in horses that are fed pasture grass, silage, and hay. The present study aims to characterize the fecal microbiota in horses that were exclusively on one of the three forage diets, and to analyze the potential impact of these forages, especially silage, on horse intestinal health. There were 36 horses randomly assigned to each of the three groups; each group was fed only one type of forage for 8 weeks. High throughput sequencing was applied to analyze the bacterial taxa in fecal samples collected from the horses at the end of the feeding trial. The *Lachnospiraceae* family was statistically more abundant in horses fed with hay, while it was the least abundant in horses fed with silage. The *Streptococcaceae* spp., considered a core microbial component in equine intestinal microbiota, were present in significantly lower quantities in feces from horses that were fed pasture grass as compared to those from horses fed hay or silage. The novel data may help promote the balancing of horse intestinal microbiota and the maintenance of intestinal health in horses.

## 1. Introduction

Horses (*Equus caballus*) are hindgut fermenters characterized by a complex microbiota, mostly comprising anaerobic microorganisms that facilitate the digestion of a high-fiber diet [1,2,3]. The intestinal microbiota also impacts the host’s immune system, influences the animal’s metabolism, and helps in the detoxification of harmful substances [4,5]. Therefore, the disruption of an animal’s intestinal microbiota can have major consequences on its overall health. The intestinal microbiota in mammals is influenced by many external factors, including lifestyle, environment [6], as well as dietary patterns [7]. The variance of microbial composition is also associated with changes in the gastrointestinal ecosystem and potentially systemic health conditions [8].

The fecal microbiota has been commonly used to study the effects of diet on the equine intestinal microbiota [7,9,10]. Diet has a significant impact on the intestinal microbiota [11]. Changes to the natural diet pattern of horses associated with domestication have influenced various bodily systems, thus causing health issues such as colic [12,13,14], metabolic syndrome [15], laminitis [16], and other dysfunctions. There is evidence that changes in dietary patterns alter the colonic microbiota, subsequently leading to changes in colonic pH and fermented products [12], and some of these changes may predispose horses to colic. Understanding the impact of different dietary patterns on the intestinal microbiota will help to reveal connections between diet and the overall health of horses.

In the present study, we investigated the effects of three different forage feeds—grass at pasture, silage, and hay—on the horse fecal microbiota. Traditionally, pastures and hay have been the major feed sources for horses [17,18]. In contrast, forages wrapped as bales and sealed in air-tight bags for anaerobic fermentation, including silage [19], have been fed to horses in some European countries as well as in Brazil [20,21,22]. These types of diet are easier to store as compared to hay and are associated with a lower emission of airborne irritants, which tend to reduce the occurrence of severe equine asthma [23]. Silage also has a lower dry matter proportion, a higher concentration of lactic acid, volatile fatty acids (VFA), and other fermented products as compared to hay. These nutrients, mainly VFA, can be readily absorbed and assimilated by the body [19]. These characteristics also make silage more digestible than hay [24]. A study has also shown that horses expressed obvious preference for silage in comparison with hay [25], which may be due to the fermentation dominated by lactic acid production and a high concentration of VFA, especially butyric acid in silage [26]. Only a few studies focused on the impact of silage on the equine hindgut ecosystem [27,28]. One study showed that no major microbial alterations were observed in fecal samples taken from horses on the silage and hay diets [27], while another one showed higher counts of *Streptococci* in colon samples taken from horses that were fed hay as compared to those from horses that were fed silage; the reason for this is still unclear [28].

In China, silage is used widely in the northwest area, such as the Xinjiang province, since silage is less expensive and easier to store as compared to other feeding materials. In other areas of China with enough pasture sources, the forage and roughage diets are more commonly used [29]. To our knowledge, there are no studies on the effects of silage on the equine fecal microbiota that used a high-throughput sequencing technique. We hypothesized that, given the significant variation in the constituents of silage as compared to grass and hay, the fecal microbial composition of horses feeding on silage might be affected and hence would differ markedly from horses that are fed other sources of forage.

The study revealed the impact of different diets on the equine fecal microbiota, which might facilitate an understanding of dietary influence on the gastrointestinal health of horses and promote research on making more appropriate adaptations for the horse diet in areas that mainly use silage in feeding.

## 2. Materials and Methods

### 2.1. Ethnic Announcement

The study was performed at the Guanzhong Stud farm in Shanxi province, China. The project was conducted with the welfare license (No.AW11101202-2-1) from the Animal Care and Use Committee of China Agricultural University.

### 2.2. Animal Selection and Experimental Design

Twenty-four mares and twelve stallions, all of the Guanzhong breed, were randomly selected from a large population at a Horse Ranch in Shanxi Province. The body weight of these horses were calculated with the estimation formula developed by Carroll and Huntington [30], and they weighed an average of 391 kg (SD ± 21.4 kg). The horses used had no history of illness and antibiotic administration in the 6 months preceding the study. During this time, the horses were found to be clinically healthy based on the farm records and physical examinations; varied in age—from 3 to 15 years old (mean age = 7 years old); and had a mean body condition score of 4.8 (SD ± 0.6), based on the Henneke scoring system. Before the study, all the horses were kept in individual stalls and fed the same commercial hay, with corn-based concentrates formulated by this farm.

The experimental dietary patterns were three forage-only diets: (1) local pasture ryegrass (September–October) (15% DM), (2) soft, dough-high stage ryegrass silage (26% DM), and (3) second-cutting ryegrass hay (91% DM) (Table 1). All the feeding grass was from the same pasture. Before the start of the feeding trial, three representative samples were collected from each type of diet. The three samples of each diet were combined for nutrition-content testing at the Animal Nutrient Analysis Center of China Agricultural University (Beijing, China). Nutritive content was measured or calculated according to the AOAC analytical methods (AOAC Int. 2010).

The mares (24) and the stallions (12) were equally divided into three groups, after which one group of mares was randomly combined with one group of stallions. As a result, three groups of horses were created, with the same male-to-female ratio. Each group was assigned one of the three dietary patterns and started the feeding trial at 8:00 am on the same day. The experiment started in September and continued for 8 weeks [31]. A week before the experiment, the diet of each horse was gradually changed by mixing new and old feed at a ratio of 25:75 for three days, 50:50 for two days, 75:25 for two days, and finally, 100% of the forage assigned to the group. For the following 8 weeks, the pasture group (group P) was maintained on the pasture of the ranch during the entire feeding trial. The silage group (group S) and the hay group (group H) were housed individually and fed three times a day throughout the trial. Horses in group S and group H were allowed 30-min paddock-based exercise each day, although grazing was restricted with the use of a muzzle. The nutrition requirement of each horse from group S and group H was calculated based on 2% of the body mass as dry matter intake for maintenance. All groups had free access to water without administering concentrate feed, other dietary supplements, or medical treatment. The body weight of each horse was monitored weekly throughout the trial in the same method descirbed presviously in this study. A physical examination of each horse was performed weekly as well to monitor their health condition.

### 2.3. Fecal Sampling

All fecal samples were collected manually from the mid-rectum of each horse at the end of the feeding trial, for three consecutive days [32,33]. Fresh samples (108 in total) were immediately saved in individual sterile plastic bags and temporarily placed in ice, for no more than 2 h. Upon arrival at the laboratory, the central part of each fecal sample was collected [34] and transferred into a sterile cryogenic vial (Corning, NY, USA) with the use of sterile Q-tips. A new Q-tip was used for each individual horse. All tubes were marked before being stored at −80 °C for further analysis.

### 2.4. DNA Preparation

Three samples of each individual were thawed and pooled together before DNA extraction. The E.Z.N.A.^®^ soil DNA Kit (Omega Bio-tek, Norcross, GA, USA) was used to extract the bacterial genomic DNA from fecal samples following the manufacturer’s instructions. After DNA extraction, the DNA concentration and purity were assessed with the NanoDrop 2000 UV-vis spectrophotometer (Thermo Fisher Scientific, Waltham, MA, USA).

### 2.5. High-Throughput Sequencing

The hypervariable region V3–V4 of the 16S rRNA gene was amplified using primer pairs 515F (5′-GTGCCAGCMGCCGCGGTAA-3′) and 806R(5′-GGACTACHVGGGTWTCTAAT-3′) [35]. The PCR product was later purified using the AxyPrep DNA Gel Extraction Kit (Axygen Biosciences, Union City, CA, USA) according to the manufacturer’s recommendations, and quantified using the Quantus Fluorometer (Promega, Madison, WI, USA). The purified amplicons were pooled in equimolar, and paired-end reads were sequenced on an Illumina MiSeq PE300 platform/NovaSeq PE250 platform (Illumina, San Diego, CA, USA) according to the standard protocols by Majorbio Bio-Pharm Technology Co., Ltd. (Shanghai, China).

### 2.6. Bioinformatics Analysis

The raw 16S rRNA gene sequencing reads were quality-filtered and merged using the FASTQ (v 0.20.0) [36] and FLASH (v 1.2.7) [37], respectively (a maximum mismatch ratio of the overlap region at 0.2, and no ambiguous nor unassembled reads were allowed). Operational taxonomic units (OTUs) with a 97% similarity cutoff [38,39] were clustered by UPARSE (v 7.1) [39]. QIIME (v 1.9.1) was applied to identify the most abundant sequence of each OTU. The RDP Classifier (v 2.2.0) [40] was used to analyze the representative sequence of each OTU taxon, which was later comparedagainst the Silva rRNA database (v 138) with a confidence threshold of 0.7.

Rarefaction curve plotting and alpha diversity analysis (Chao and Shannon indexes) were performed by MOTHUR (v 1.3.0) (Ann Arbor, MI, USA). The Student’s t-test was applied for the comparison of the alpha diversity difference between groups. A principal coordinates analysis (PCoA) based on the Bray–Curtis distance was generated using QIIME (v 1.9.1) [41] to assess the bacterial community composition between groups. Analysis of Similarity (ANOSIM) was applied to test the significance of clustering associated with diet. A non-parametric factorial Kruskal–Wallis sum-rank test was used in a Linear discriminant analysis effect size (LEfSe) [42] to detect significant differences of microbial taxa between the three groups.

## 3. Results

The body condition score of all horses did not change and their body weight remained relatively stable throughout the feeding trial. Throughout the trial, the physical examination was unremarkable for each horse, and no clinical abnormalities were observed.

### 3.1. Sequencing Quality Data

A total of 2,463,742 sequence reads were detected from all the samples, while 1,181,628 sequences—ranging from 32,823 to 71,449 per sample—remained after quality control. After clustering at the 97% threshold level, 3644 OTUs were retained and classified as bacteria, with 29 phyla, 64 classes, 142 orders, 263 families, and 530 genera.

**Figure 1:**
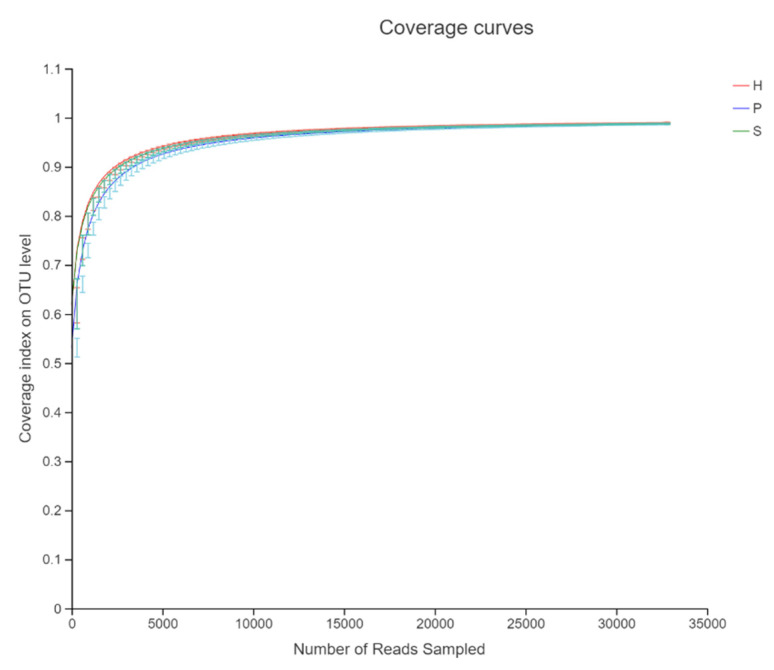
Asymptotes of the rarefaction curve (coverage index) compares sequencing depth between three dietary patterns.

The rarefaction curve for the number of observed species indicated adequate sequencing depth. It also showed an almost full coverage of the bacterial community from sampling (Figure 1).

### 3.2. Microbial Composition Analysis

**Figure 2:**
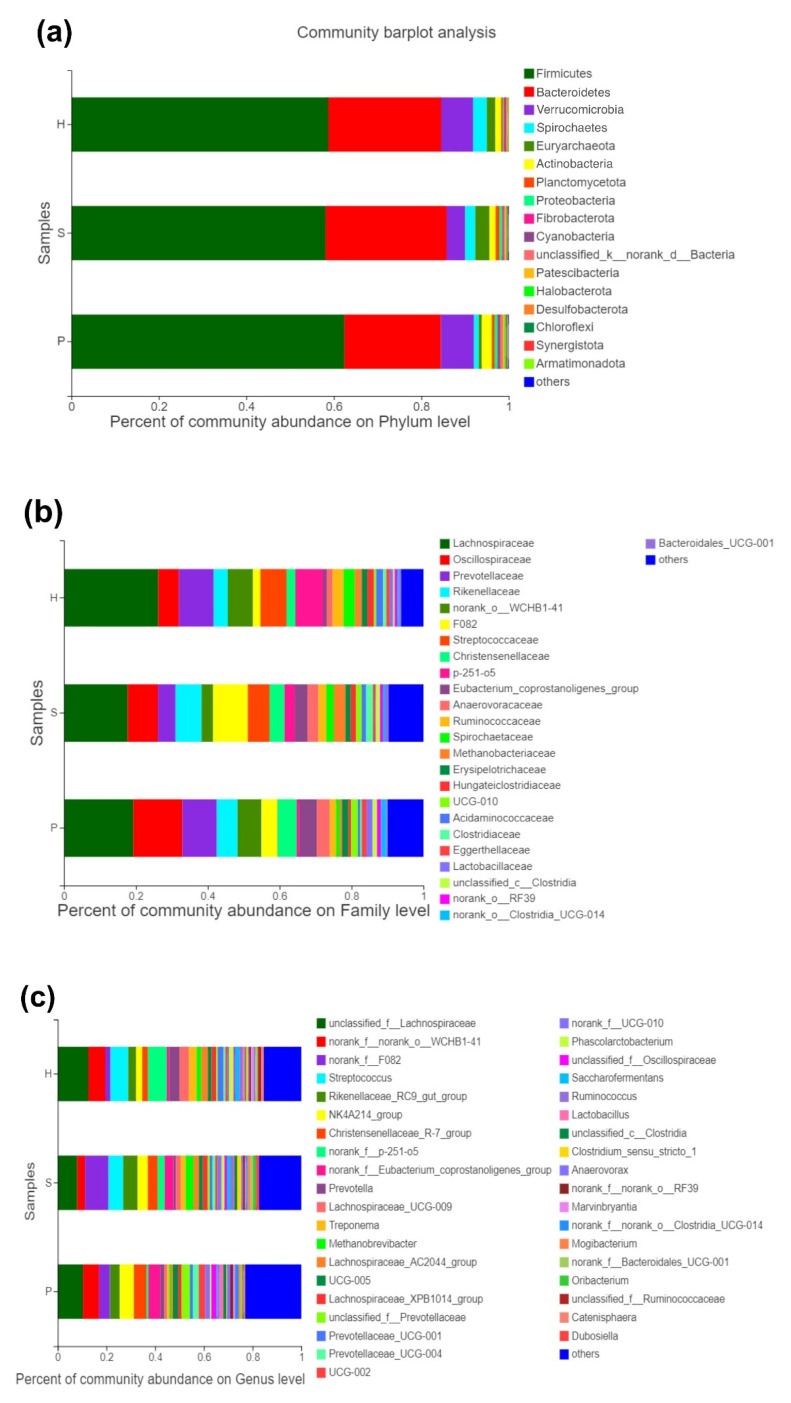
Relative abundance of the dominant bacterial community at the phylum (**a**), family (**b**), and genus (**c**) levels of the three groups. The Student’s *t*-test was used to assess the difference between the alpha diversity of the groups of samples.

Stacked histograms were used to illustrate the relative abundance of the dominant bacterial population at the phylum, family, and genus levels (Figure 2). Six phyla, twenty-five families, and thirty-eight genera had a mean relative abundance of more than 1% and were illustrated in histograms. Firmicutes (59.8%), Bacteroidetes (25.2%), Verrucomicrobia (6.4%), Spirochaetes (2.2%), Euryarchaeota (1.9%), and Actinobacteria (1.7%) were the most abundant phyla in all groups (Figure 2a). Additionally, the relative abundance of Verrucomicrobia, Spirochaetes, Euryarchaeota, and Actinobacteria differed significantly (*p* < 0.05) between the groups.

At the family level, *Lachnospiraceae* and *Oscilliospiraceae* were the top two abundant bacterial taxa in all groups, which rendered a significant difference between the groups (*p* < 0.01) (Figure 2b). *Lachnospiraceae*, belonging to the phylum Firmicutes and the order Clostridiales, had the highest relative abundance in group H, while the lowest in group S. *Oscilliospiraceae*, which also belong to the phylum Firmicutes and the order Clostridiales, were most abundant in group P. *Streptococcaceae*, a member of the phylum Firmicutes and the order Lactobacillales, ranked seventh based on the average relative abundance in all groups and demonstrated a tremendously low level in group P (*p* < 0.001).

At the genus level, seven genera that could not be ranked or classified were abundant in all three groups. Most of the genera were detected as significantly different between all groups (Figure 2c). *Streptococcus* were the fourth most abundant taxa in all groups at this level and revealed a significantly lower relative abundance in group P (*p* < 0.001).

### 3.3. Alpha Diversity Analysis

**Figure 3:**
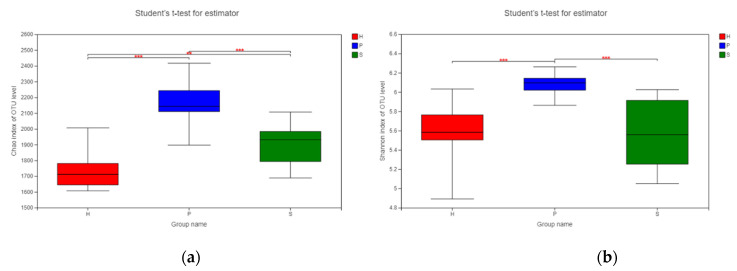
Alpha diversity indices of the fecal microbiota among horses on pasture grass (P, n = 12), silage (S, n = 12), and hay (H, n = 12) dietary patterns. ** *p* ≤ 0.01, *** *p* < 0.001. (**a**) Bacterial species richness or Chao index per group of samples. (**b**) Bacterial species diversity or Shannon index per group of samples. Red: group H; blue: group P; green: group S.

An alpha diversity analysis was conducted with the Chao index and the Shannon index to analyze the microbial species richness and diversity among the three groups in different dietary patterns (Figure 3). Notable differences in the Chao index were identified among all three groups (Figure 3a). Group P had the highest score from the Chao index, representing the richest microbial community among the three groups, while group H had the lowest OTU richness. The microbial diversity of the group S and group H samples were significantly lower than that of group P, as revealed by their lower Shannon indexes (both *p* < 0.001) (Figure 3b).

### 3.4. Beta Diversity and LEfSe Analysis

**Figure 4:**
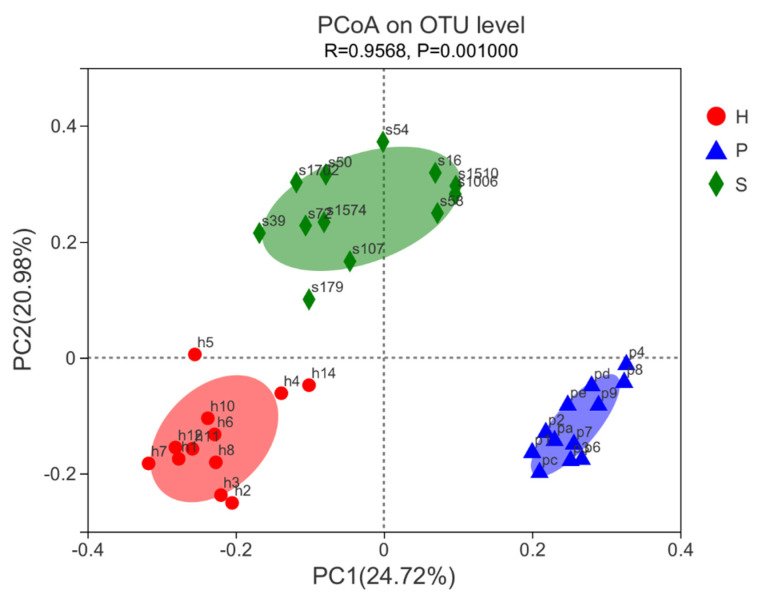
Principal coordinates analysis (PCoA) of Bray-Curtis distances illustrates proximity matrices present in feces of horses with three various dietary regimens after 8 weeks of feeding trial. Red: group H; blue: group P; green: group S.

A beta diversity analysis of samples was applied to evaluate differences of fecal bacterial communities in the three groups. The PCoA analysis based on the Bray–Curtis distance was performed (Figure 4). The bacterial composition of each group clustered notably at the OTU level according to the dietary regimens. Group S showed a slightly higher variability compared with those of group P and group H.

**Figure 5:**
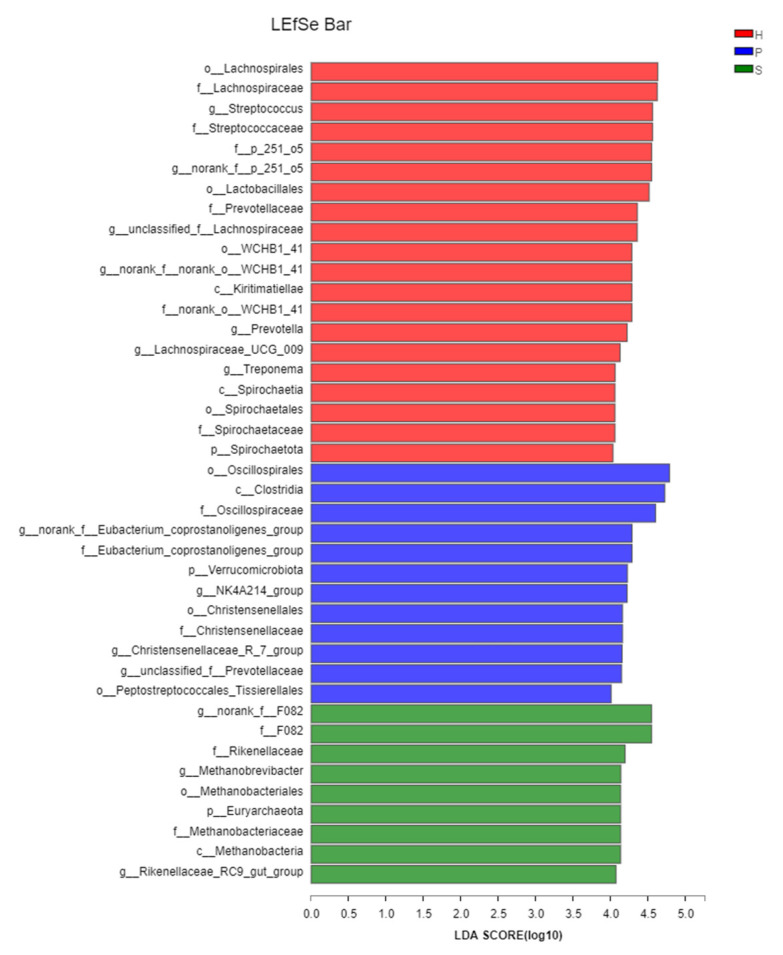
LEfSe discriminates difference of the fecal microbiota among group H, group P, and group S. 414 taxa were statistically different between groups (*p* < 0.05), and only taxa with an LDA score of 4 or above were shown and ranked by the effect size in LEfSe. Red: group H; blue: group P; green: group S.

A linear discriminant analysis (LDA) Effect Size (LEfSe) was applied to identify the discriminatingly abundant taxa of microbial populations across the feeding patterns. From 505 genera or higher taxa, 414 taxa were statistically different among the three groups (*p* < 0.05). Of these, 26 had an LDA score with the threshold > 3.0. There were 6 out of 26 taxa associated with group S, while seven and thirteen taxa were associated with group P and group H, respectively (Figure 5). In the samples of group S, the most distinct bacterial taxa were F082, *Rikenellaceae*, and Methanobacteriales, while Oscillospirales, Clostridia, and *Oscillospiraceae* were the three taxa with the highest effective size in group P. Lachnospirales, *Lachnospiraceae*, and *Streptococcaceae* were the top three most associated with group H.

## 4. Discussion

The microbial community in the gastrointestinal tract of horses was shaped by many factors [43]. Therefore, in this study, the feeding trial was started at the same time on the same day, and maintained consistently for 8 weeks to ensure adaptation to new diets in selected horses [27]. Furthermore, male and female horses have significantly different fecal microbiota at each level of classification [44]. Hence, this study maintained the same ratio of mare to stallions (2:1) in each group to standardize any sex effect. Obesity and age are associated with fecal microbial changes [45]. There is evidence that aged horses (>19 years old) might be more predisposed to metabolic diseases and changes of body fat mass, with subsequent influences on the intestinal microbial composition [45]. In the current study, horses were selected based on their similar body condition scores (BCS 4–6), with ages between 3 and15 years old, to decrease microbiota variance related to obesity and aging. Horses in groups S and H were fed their respective forage daily, at a dry weight equivalent to 2% of each horse’s body mass, which is suitable for the majority of animals [46]. However, the amount of pasture grass consumed was not easy to estimate. It has been reported that the daily voluntary intake of dry matter by a horse is about 2.6% of their body mass [18], which can help with the DM estimation of group H. In addition, the BCS and body weight of each horse were monitored throughout the trial to ensure that no significant changes occured in the two factors.

Greater changes were demonstrated in fecal bacterial diversity and structure associated with different diets, especially with supplementation. Therefore, it is considered a potentially good indicator while studying the equine intestinal ecosystem [47]. Other advantages of using fecal samples include easy collection, reliable dysbiosis detection, and convenient microbial tracking [48]. In the current study, significant differences of richness and diversity of fecal microbiota were demonstrated among the three groups. Horses maintained on pasture had the richest and most diverse OTUs, while microbiota in group H had the least OTU richness and diversity. There is evidence that a diverse community of organisms is critical to the maintenance of healthy microbiota and resillience to the overgrowth of pathogenic microbial floras [49]. The OTU results in group P probably indicate that higher richness and diversity of intestinal microbiota could be an evolutionary strategy for herbivorous browsers to stay healthy while dealing with the unstable quantity and quality of feed available under the circumstance of naturally grazing on pasture [50]. In humans, decreases in microbial diversity and richness have been associated with some GIT disorders such as chronic diarrhea [51]. In horses, it has also been observed in colic patients presented to the hospital [52]. The less rich and diverse OTUs of group H might not indicate an abnormality since the horses were clinically healthy. An analysis of the fecal microbiota prior to the feeding trial can be helpful to determine if it is a significant alteration.

The microbial composition was statistically different between the groups, as illustrated by the PCoA plot with three distinct clusters (Figure 4). Silage is fermented anaerobically from forage to facilitate preservation, and has been used as one of the main diets for horses in China [29] and other countries [53]. Studies exploring the association of equine fecal microbiota with the silage diet are scarce [27]. We hypothesized that silages characterized with higher acidity and as more fermented products than hay and fresh grass may exert a distinct impact on the hindgut microbiota of horses. In the current study, horses that were fed silage had the highest relative abundance of Euryarchaeota, while horses that were fed pasture grass had the lowest. This trend was consistent with the levels of NDF and ADF measured in the forages. Phylum Euryarchaeota contains all presently known methanogen [54], and there is a correlation between methane output—one of the fermented products—and the concentration of fiber in the diet [55], especially ADF, which is not easily digestible [56].

Firmicutes and Bacteroidetes have consistently been reported as the predominant proportion of fecal and hindgut microbiota in healthy horses [45,57,58,59], whereas a marked decrease of Firmicutes and an increase of Bacteroidetes have been noted in horses with colitis [58]. These two phyla were the most abundant in all three groups without any significant differences of relative abundance between groups, which indicated their overall healthy intestinal status. Fibrobacteres, the fibrolytic phylum, is commonly abundant in equine intestinal microbiota [7,35,60,61]. Several studies such as those by Shepherd et al. (2012), Costa et al. (2012), and Daly et al. (2012) [10,58,62] reported low abundance, although the cause of the varying abundance of Fibrobacteres in equine intestinal microbiota remains unclear. In this study, it had a low relative abundace in all groups (0.4% in group H, 0.3% in horses fed silage, and 0.07% in group P) and its level positively correlated with the dry matter content in the three forages. Further research is necessary to determine the relationship between the relative abundance of Fibrobacteres and different diets.

The *Lachnospiraceae* was the bacterial family with the highest relative abundance in all three groups, while it also varied significantly between groups. It was the most abundant in horses that were fed hay, and the least abundant in the silage group. The *Lachnospiraceae* consists of a large and diverse cluster of fibrolytic and saccharolytic bacteria such as *Clostridium* spp., *Ruminococcus* spp., and *Eubacterium* spp. [62]. There is also evidence that many bacteiral members in the *Lachnospiraceae* family possess the ability to express different genes that are associated with fibrolytic and saccharolytic digestion [63]. There are also abundant and diverse genes to express carbonhydrate-active enzymes in this family that help with the digestion of fibrous components in plants [63,64]. In the present study, hay comprised the highest dry matter content, according to Table 1; therefore, it might contribute to the increase of fibrolytic bacteria in the hindgut ecosystem to facilitate fiber digestion in horses that are fed hay.

The *Lachnospiraceae* has been found in the gastrointestinal tract (GIT) of many mammals [63]. In humans, the *Lachnospiraceae* have demonstrated the ability to convert lactate to butyrate [65], which is critical in the maintenance of healthy intestines and the reduction of the risk of intestinal inflammation. The *Lachnospiraceae* is also involved in the production of short-chain fatty acids (SCFA), which act as growth factors for healthy epithelium [65,66]. Another study in humans showed that low levels of this family might be correlated with inflammatory bowel diseases [67], which also strengthened its importance in the health of GIT. In the current study, the *Lachnospiraceae* was least abundant in horses that were fed silage, which might impact the ability of butyrate conversion. Further investigation is necessary to look at whether the decrease of *Lachnospiraceae* in the intestinal microbiota is correlated with a compromised GIT health of horses that are fed silage in the long term.

Among all three groups, the *Oscillospiraceae* was another bacterial family with significantly different levels between groups. It was the most abundant in horses managed on pasture and the least in group H, which indicated that its presence could have been influenced by different diets. Based on the result of LEfSe, Oscillospirales and Oscillospiraceae were more associated with group P. Certain ingredients of the diet might exert influence on the relative abundance of *Oscillospiraceae*, such as crude protein, which was found to be most abundant in pasture grass in this study. However, further investigation is necessary to promote the understanding of the relationship between *Oscillospiraceae* and diet, as well as its role in intestinal health.

The *Streptococcaceae* was the bacterial taxon most affected by dietary patterns in this study. The OTUs detected in horses from group P was tremendously lower than those in the other two groups. There was a similar trend for *Streptococcus* at the genus level. The *Streptococcaceae* is under the order Lactobacillales and includes some lactic-acid producing bacteria, for example *Streptococci* [68]. The *Strepcoccoaceae* has been detected in some studies as one of the main bacterial taxa in the healthy horse hindgut ecosystem [69,70], because some species have lost the virulent factors and genes during evolution and adapted to the ecosystem of animals [71]. However, the proliferation of some *Streptococcus* spp. has been strongly associated with the onset of laminitis induced by carbohydrates [72]. It has also been reported to elevate significantly when horses are fed a high-startch diet. However, it is still unknown whether the increase has any wider health implications for the horses [60]. Further analysis of carbohydrate content in the forages involved in this study may help investigate its association with the low abundance of *Streptococcaceae* in group P. *Streptococci* has been reported absent in horsess involved in some studies [45,50]. One of the studies reported that no *Streptococcus* was detected in clinically healthy horses fed on pasture in New Zealand [50], which was similar to our result of extremely low levels of *Streptococcus* in horses managed on pasture. The fecal microbiota in the horses prior to the feeding trial might provide more clues as to whether the low level of *Streptococcaceae* in group P was associated with diet change. It was not detected in this study since the main objective was to compare the fecal microbiota of horses on three exclusive diets.

The most distinct bacterial taxa in horses that were fed silage were F082, *Methanobacteriales*, and *Rikenellaceae*. Studies on these taxa were still limited in reporting its presence, especially of the newer taxa F082 and *Rikenellaceae*; therefore, their roles in intestinal microbiota are still undetermined [73,74]. As a result, more research, including functional analysis, is required to help understand the relationship between silage feeding and its associated fecal bacterial taxa.

Finally, at the genus level, the seven unranked or unclassified genera were abundant in all groups. This reflected our overall lack of knowledge on the equine intestinal ecosystem. Therefore, other bacterial identification methods could be involved to promote further investigation on the equine intestinal microbiota.

## 5. Conclusions

In this study, the fecal microbiota of horses was found to have distinct characteristic patterns in horses on different diets. For the first time, the baseline information on the fecal microbiota of horses that were fed with silage was established. It was revealed that *Lachnospiraceae* was least abundant in horses that were fed silage, compared with the other two groups, which was a significant finding as it may indicate inflammatory changes as revealed by previous studies on humans. Other than that, the silage diet did not generate other apparent dysbiosis on the equine fecal microbiota in comparison with the other two common forages of horses. More studies are warranted to further define the impact of the silage diet on equine intestinal health.

## Figures and Tables

**Table 1:** Chemical composition: pasture grass, silage, and hay (as fed).

	Pasture Grass	Silage	Hay
DM ^1^ (%NM ^2^)	15.53	25.78	91.50
CP ^3^ (%DM)	13.11	8.54	9.36
CF ^4^ (%DM)	0.98	1.34	1.82
Ash (%DM)	11.24	9.91	5.04
NDF ^5^ (%DM)	47.56	66.62	59.89
ADF ^6^ (%DM)	29.02	46.94	34.71
Ca ^7^ (%DM)	0.57	0.28	0.16
P ^8^ (%DM)	0.13	0.22	0.06

^1^ dry matter; ^2^ natural matter; ^3^ crude protein; ^4^ crude fat; ^5^ neutral detergent fiber; ^6^ acid detergent fiber; ^7^ calcium; ^8^ phosphorus.

## Data Availability

The data presented in this study are openly available in FigShare at 10.6084/m9.figshare.14551497.
